# Standardized amino acid digestibility and nitrogen-corrected true metabolizable energy of frozen raw, freeze-dried raw, mildly cooked, and retorted dog foods using the precision-fed cecectomized and conventional rooster assays

**DOI:** 10.1093/jas/skaf334

**Published:** 2025-09-27

**Authors:** Elizabeth L Geary, Christina L Vogel, Carl M Parsons, Pam L Utterback, James R Templeman, Mary-Grace C Danao, Kelly S Swanson

**Affiliations:** Department of Animal Sciences, University of Illinois Urbana-Champaign, Urbana, IL 61801; Department of Animal Sciences, University of Illinois Urbana-Champaign, Urbana, IL 61801; Department of Animal Sciences, University of Illinois Urbana-Champaign, Urbana, IL 61801; Department of Animal Sciences, University of Illinois Urbana-Champaign, Urbana, IL 61801; Primal Pet Foods, Primal Pet Group, Fairfield, CA 94534; Food Science and Technology Department, University of Nebraska-Lincoln, Lincoln, NE 68583; Department of Animal Sciences, University of Illinois Urbana-Champaign, Urbana, IL 61801; Division of Nutritional Sciences, University of Illinois Urbana-Champaign, Urbana, IL 61801; Department of Veterinary Clinical Medicine, University of Illinois Urbana-Champaign, Urbana, IL 61801

**Keywords:** canine nutrition, diet processing, pet food

## Abstract

The application of heat in dietary processing is known to influence nutrient digestibility. Novel pet food formats with differing processing methods are gaining popularity, but few studies have examined their digestibility. Most research evaluating dietary processing type on nutrient digestibility has tested commercial foods that were vastly different regarding ingredient inclusion and macronutrient content, making it difficult to determine the processing influences. To address this research question, the current study aimed to determine amino acid (AA) digestibility and nitrogen-corrected true metabolizable energy (TME_n_) of diets having the same ingredient formulations and nutrient concentrations but manufactured using different processing methods. Five diets were manufactured using the following processing methods: retort (RT), mildly cooked [sous vide (SV) and steamed (ST)], and raw [high-pressure processing (HPP) and freeze-drying (FD)]. Those diets were compared against the raw ingredient batch (RAW) that served as a control. Two precision-fed rooster assays utilizing Single Comb White Leghorn (1.5 to 2.5 y old, 2.5 to 3 kg body weight) were conducted to determine the standardized AA digestibility (30 cecectomized roosters; *n* = 5) and TME_n_ content (30 conventional roosters; *n* = 5) of the 6 pet foods. Prior to feeding, wet diets (RT, SV, ST, HPP, and RAW) were freeze-dried, and all diets were ground. Following crop intubation, excreta were collected for 48 h and analyzed, and then AA digestibility and TME_n_ calculations were performed. Data were analyzed using the Mixed Models procedure of SAS with *P *< 0.05 accepted as statistically significant and *P *< 0.10 a trend. The digestibility of 6 indispensable AA were affected by processing. The SV and ST diets had greater (*P *< 0.05) histidine digestibilities than all other diets. For valine, methionine, leucine, phenylalanine, and isoleucine, the RAW diet tended to have greater (*P *< 0.10) digestibility than the RT diet. The RT diet had lower (*P *< 0.05) aspartic acid digestibility than ST, HPP, FD, and RAW diets. Dietary TME_n_ was higher (*P *< 0.05) for the SV and ST diets than the RT, HPP, and FD diets, suggesting that those cooking methods are less damaging to macronutrients. Overall, the RT diet had lower indispensable digestible AA concentrations than RAW, likely due to the high heat of processing. Future research should test differences in these diet types in the target species (i.e., dog) to evaluate how they perform.

## Teaser

The current study used the cecectomized rooster model to evaluate the amino acid digestibilities and energy content of pet foods made from the same ingredient inclusions and macronutrient targets but processed with 6 distinct methods.

## Introduction

The temperature and duration of heat application is known to influence nutrient, particularly protein, digestibility (Hendriks et al., 1999; Williams et al., 2006; Almeida et al., 2013; Oba et al., 2019). Traditional methods of pet food manufacturing, namely extrusion and retort, apply high temperatures (typically 125–150 °C and 120–127 °C, respectively) in processing (Rokey et al., 2010; Hagen-Plantinga et al., 2017). Consequently, novel pet food formats that use lower temperatures in processing are gaining popularity.

Mild (<100 °C) thermal processes can increase protein digestibility, whereas more intensive heat treatments (>100 °C) can cause proteins to aggregate, oxidize, amide bond, disulfide-link, or cross-link, which blocks digestive protease access and decreases digestibility (Bhat et al., 2021a). The extent and mechanism of protein denaturation is dictated by the peak temperature achieved, the rate of heating, holding times, and the specific amino acid (AA) residues (Estévez and Luna, 2016; Qian et al., 2017; Estévez and Xiong, 2019).

Retort is a common high-heat cooking method of manufacturing wet/canned pet foods. However, the high heat treatment of retort can cause a reduction in protein quality (determined by the composition and digestibility of AA) (Hendriks et al., 1999), with digestibility decreasing with increasing retort temperatures (Cao et al., 2024). To preserve the digestibility of proteins while still keeping foods safe for consumption, mild thermal processes are utilized in the pet food industry.

Steaming is one mild cooking method that causes the partial unfolding of proteins, such a conformational changes and mild oxidation, allowing proteases to have greater access to hydrolytic sites (Bhat et al., 2021a). Ham that had been steam cooked to a core temperature of 65 °C had increased *in vitro* digestibility compared with raw meat (Rakotondramavo et al., 2019). Sous vide cooking causes secondary structure protein unfolding from the mild heating, which enables increased exposure of proteases to hydrolytic sites. In addition to protein unfolding, endogenous enzymatic proteolysis occurs in combination with the creation of a modified microstructure, contributing to better oxidative stability and improved digestibility (Bhat et al., 2021b). In *in vitro* studies, sous vide cooking at temperatures ranging from 58 to 60 °C increased protein digestibility compared with meat cooked in a water bath at 80 °C and meat cooked in an oven at 160 °C (Kehlet et al., 2017; Bhat et al., 2020). However, there was no difference in digestibility between beef grilled at 240 °C and sous vide cooked at 80 °C when fed to humans (Prodhan et al., 2020). Kehlet et al. (2017) observed a decrease in digestibility with increased holding time (the amount of time the meat is held in the water bath after reaching a specific core temperature), whereas Bhat et al. (2020) reported the opposite effect.

Non-thermal processing, such as high-pressure processing (HPP) and freeze-drying (FD), can be utilized for reducing pathogen loads, extending shelf life, or reducing water activity while not inducing the negative impacts of high heat processing. The tertiary and quaternary structures of protein folding can denature and aggregate when exposed to pressure, but secondary structures that are stabilized by hydrogen bonds are stable up to 700 MPa (Esteghlal et al., 2019). Pressure can cause water to enter the hydrophobic interior of proteins causing unfolding, but the protein can sometimes refold once the pressure is removed (Bolumar et al., 2016). High-pressure processing at 200 MPa causes a change in structure that may be reversible, but pressure over 400 MPa causes additional hydrogen bonding, leading to increased protein aggregation, which decreases solubility (Bolumar et al., 2016; Grossi et al., 2016). Solubility of sarcoplasmic and myofibrillar proteins, generally correlating to digestibility, decreases with an increase in pressure (Chapleau et al., 2003; Lee et al., 2007; Marcos et al., 2010; Chan et al., 2011; Grossi et al., 2012; Speroni et al., 2014). However, in addition to amount of pressure, the extent of denaturation depends on a plethora of factors such as duration of application, temperature, and pH (Grossi et al., 2016). Depending on the specific protein structure and processing parameters, pressure-induced structural changes can vary (Khalid et al., 2023), which in theory could cause a decrease or increase in protein digestibility. Several *in vitro* studies have demonstrated an improvement in digestibility of meat after HPP, including Kaur et al. (2016; bovine *longissimus dorsi* muscle meat processed at 175 and 600 MPa), Rakotondramavo et al. (2019; cooked ham processed at 500 MPa for 5 min), Xue et al. (2020; rabbit *longissimus dorsi* muscle meat processed at 100, 200, and 300 MPa for 9 min), and Cepero-Betancourt et al. (2020; red abalone muscle processed for 200, 300, 400, and 500 MPa for 5 min). This improved digestibility is possibly due to myofibrillar disorganization and modification of protein structures, exposing more cleavage sites. Freeze-drying can also have varying effects on protein digestibility (Wong and Cheung, 2001; Deng et al., 2015; Sá et al., 2019; Lee et al., 2024), being largely dependent on specific temperature and time parameters (Lee et al., 2024).

Given the impacts of high heat processing and pressure on proteins, mildly cooked and raw pet foods are believed to have greater AA digestibilities (an important factor in determining protein quality) than retorted pet food. Therefore, the objectives of this study were to determine the AA digestibility and nitrogen-corrected true metabolizable energy (TME_n_) of frozen raw, freeze-dried raw, mildly cooked, and retorted dog diets containing the same ingredient inclusion levels and nutrient concentrations. We hypothesized that the frozen and freeze-dried raw diets would have greater AA digestibilities than the mildly cooked and retorted diets, but the mildly cooked diets would have greater AA digestibilities than the retorted diet. Additionally, we hypothesized that the TME_n_ will follow the same pattern due to the differences in digestibility.

## Materials and Methods

All animal care procedures were approved by the University of Illinois Urbana-Champaign Institutional Animal Care and Use Committee prior to the start of the experiment (IACUC #23092).

### Diets

All diets were formulated to meet the Association of American Feed Control Officials (AAFCO, 2024) nutrient profiles for adult dogs at maintenance (Table 1). One retorted (RT) diet, 2 mildly cooked diets [one processed using sous vide (SV) cooking; another using steam (ST) cooking], one high-pressure processed raw (HPP) diet, one freeze-dried (FD) diet, and the raw batch mix (RAW) were tested in this study.

**Table 1.** Dietary formulas of experimental diets

Ingredient	RT	SV, ST, HPP, FD, RAW
	--- %, as-is ---	
**Chicken, mechanically separated**	43.931	44.063
**Chicken, liver**	23.962	24.034
**Chicken, neck**	19.969	20.029
**Carrots**	2.995	3.004
**Squash**	2.995	3.004
**Egg, dried**	1.4975	1.502
**Flax seed**	1.4975	1.502
**Fish oil**	0.599	0.601
**Coconut oil**	0.599	0.601
**Miscanthus grass**	0.599	0.601
**Calcium carbonate**	0.500	0.501
**Sunflower oil**	0.360	0.361
**Guar gum**	0.300	0.000
**Vitamin premix^1^**	0.087	0.087
**Potassium chloride**	0.050	0.050
**Rosemary extract**	0.040	0.040
**Mineral premix^2^**	0.020	0.020

1 Provided per kg diet: 45.15 mg Ca as calcium carbonate, 59.92 IU vitamin E, 15.40 mg niacin, 10.53 mg pantothenic acid (as d-calcium pantothenate), 6.20 mg riboflavin, 8,224.16 IU vitamin A, 3.09 mg thiamine (as thiamine mononitrate), 2.25 mg pyridoxine (as pyridoxine hydrochloride), 27.58 mcg vitamin B-12, 568.96 IU vitamin D, and 322.23 mcg folic acid.

2 Provided per kg diet: 18.14 mg Fe (as FeSO_4_), 20.62 mg Zn (as ZnSO_4_), 26.44 mg K (as KCl), 3.10 mg Cu (as CuSO_4_), 72.00 mcg Se (as Na_2_SeO_3_), 1.57 mg Mn (as MnSO_4_), and 390.40 mcg I [as Ca(IO_3_)_2_].

All diets were based on the same ingredient formula, with chicken (mechanically separated, liver, and neck) comprising almost 90% of the formulation. There was a slight difference in the RT diet, being the inclusion of guar gum as a hydrocolloid (Table 1). Primal Pet Group (Fairfield, CA) sourced all raw ingredients. After mixing ingredients and freezing diet mixtures, they were shipped to The Food Processing Center at the University of Nebraska-Lincoln (Lincoln, NE) for manufacturing, except for the HPP diet which was cold pasteurized at an HPP tolling facility in Lincoln, NE. Diets were processed using the procedures listed below.

To process the RT diet, 0.3% guar gum was first added to the raw mix and mixed for 5 min in a ribbon mixer. Retort pouches were then filled with 200 ± 5 g and vacuum sealed. Pouches were stored in a walk-in cooler (3–4 °C) prior to being cooked at 122 °C for 14 min (including a minimum sterile 1 time of 2 min for the wet down level to reach 90%, 30 s of stabilization, and 11 min of remaining cook) and cooled for 25 min using a pilot-scale FMC FoodTech Log-Tec momentum retort cooker (Philadelphia, PA). This temperature-time combination was based on preliminary tests that showed the internal temperature of the coldest pouch inside the retort had a come up time of 7–9 min, depending on the number of pouches loaded inside the retort. The pouches needed to be held at 122 °C for 3 min in order to achieve a 12-log reduction in *Clostridium botulinum* spores, thereby sterilizing the product (Anderson et al., 2011). Processing temperatures and internal temperatures were monitored via probes during the entire process. The two mildly cooked diets (SV and ST) were processed by sous vide and steaming, respectively. Specifically, the raw diet mix was heated with sous vide cookers (Inkbird, Shenzhen, China; Anova Culinary, San Francisco, CA) in 4.55 kg chubs in a water bath set to 84 °C. The temperature never exceeded 85 °C and the chubs were cooked for 4 h, achieving an internal temperature of 74 °C (validated via internal temperature probes). The bath temperature setting was used to ensure that the total time the product temperature was between 10 and 54 °C during the come up time was 6 h or less and the final internal temperature assured a 7-log reduction in *Salmonella* (USDA FSIS, 2021). For the ST diet, the raw diet mix was processed in a smokehouse (Enviro-Pak, Portland, OR) in 4.55 kg chubs using only steam (no smoke) in stages [49 °C for 1 h; 60 °C for 1 h; 85 °C until a core temperature of 74 °C was reached (validated via internal temperature probes)] (USDA FSIS, 2021). In the first 3 stages, steam was maintained at a relative humidity of 85%. During the last stage, the diet was cooled at 16 °C for 30 min with no added steam. To manufacture the HPP diet, the raw diet mix was processed in 4.55 kg chubs using high pressure (600 MPa; 87,000 psi) (Avure, Middletown, OH) for 3 min using chilled water (3–4 °C) as the pressurizing fluid. These are commonly used HPP parameters in raw pet food diets to control for *Salmonella*, *Listeria monocytogenes*, and *Escherichia coli* O157:H7 and other Shiga toxin-producing *E. coli* (Lee et al., 2023). For the FD diet, the raw diet mix first underwent cold pasteurized HPP using the parameters and equipment listed above. This was done because it is a common procedure for freeze-dried foods to control *Salmonella*. The product was then diced into approximately 1.95 cm^3^ cubes and stored frozen in a −20 °C walk-in freezer prior to freeze drying in a Parker 2 Freeze Dryer (Beresford, SD). The product was first held at −40 °C for 15 min to ensure all moisture in the product was in the solid phase (ice). Sublimation was initiated by raising the shelf temperatures to 27 °C for 1 h and then to 43 °C for 5 h. Desorption proceeded using shelf temperatures of 74 °C for 10 h, followed by 63 °C for 8 h. Lastly, the RAW diet pertains to the raw diet mix with no processing prior to the freeze-drying that was necessary to crop intubate the roosters.

### Animals, housing, and experimental timeline

All birds were housed individually in cages (27.9 cm wide × 50.8 cm long × 53.3 cm high) with raised wire floors and kept in an environmentally controlled room (approximately 23.9 °C, 17 h light: 7 h dark). Two precision-fed rooster assays utilizing Single Comb White Leghorn (1.5 to 2.5 y old, 2.5 to 3 kg body weight) were conducted as described by Parsons (1985) to determine the standardized AA digestibility and TME_n_ content of the 6 pet foods. In the first rooster assay (to determine AA digestibility), 30 cecectomized roosters were randomly assigned to the foods (*n* = 5 roosters per test diet evaluated). Prior to the study, cecectomy was performed on roosters under general anesthesia according to the procedures of Parsons (1985). Roosters were given at least 8 wk to recover from surgery before being used in the experiment. In the second rooster assay (to determine TME_n_), 30 conventional roosters were randomly assigned to the foods (*n* = 5 roosters per test diet evaluated). Before the start of the experiments, feed and water were supplied for ad libitum consumption. In both assays, after 26 h of feed withdrawal but ad libitum water, roosters were tube-fed 12 g of the test substrates combined with 12 g of corn. Prior to feeding, wet diets (RT, SV, ST, HPP, and RAW) were freeze-dried (Dura-Dry MP microprocessor-controlled freeze-dryer; FTS Systems, Stone Ridge, NY), and then all diets, including FD diet, were ground through a 2-mm screen (Wiley mill model 4, Thomas Scientific, Swedesboro, NJ). Freeze-drying of the wet diets was necessary in order to achieve the correct flow to crop intubate the roosters.

### Sample collection and analysis

Following crop intubation, excreta was collected for 48 h on plastic trays placed under each individual cage. Excreta samples were lyophilized, weighed, and ground through a 0.25 mm screen prior to analysis.

### Chemical analyses

All diets, including the FD diet, were then ground in a Wiley mill (model 4, Thomas Scientific, Swedesboro, NJ) through a 2-mm screen. Diets and rooster excreta were analyzed for dry matter (DM) and ash according to the Association of Official Analytical Chemists (AOAC, 2006; methods 934.01 and 942.05), with organic matter being calculated. Crude protein for diets and excreta was calculated from a Leco Nitrogen/Protein Determinator (TruMac N, Leco Corporation, St Joseph, MI) and total nitrogen values according to AOAC (2006; method 992.15). Gross energy content of diets and excreta from conventional roosters was measured using an oxygen bomb calorimeter (model 6200, Parr Instruments, Moline, IL). For diets and excreta from cecectomized roosters, AA, including reactive and total lysine, were measured at the University of Missouri Experimental Station Chemical Laboratories (Columbia, MO) according to AOAC (2006; method 982.30E). Total lipid content of the diets and conventional excreta were determined using acid hydrolysis and extraction methods facilitated by ANKOM Technology equipment (Hydrolysis System, XT15 Extractor, and RD Dryer; Macedon, NY; AOCS Official Procedure Am 5-04). Total dietary fiber content of diets was determined according to Prosky et al. (1992).

### AA digestibility calculations

Basal endogenous AA concentrations were determined using roosters that were fasted for 48 h, and then standardized AA digestibility values were calculated by the method of Engster et al. (1985) using the equations below.


$$AA\;\mathrm{digestibility}\;of\;\mathrm{diet}\;(\%)=\;[\frac{AA\;\mathrm{consumed}-AA\;\mathrm{excreted}\;by\;\mathrm{fed}\;\mathrm{birds}+AA\;\mathrm{excreted}\;by\;\mathrm{fasted}\;\mathrm{birds}}{AA\;\mathrm{consumed}}]x\;100$$

Where AA consumed (g) = diet intake (g) × AA in diet (%); AA excreted by fed birds (g) = excreta output (g) × AA in excreta (%); AA excreted by fasted birds = excreta output (g) × AA in excreta (%). The AA digestibility values for test diets were then calculated by difference using the equation:


AA digestibility of test diet (%)=AA digestibility of ground corn reference diet -[(standardized AA digestibility of ground corn reference diet-standardized AA digestibility of test diet mixture with corn)proportion of test diet AA substituted into the test diet mixture with corn]

### Fat digestibility calculations

Conventional roosters were also used for apparent fat digestibility calculations. Apparent fat digestibility values were calculated using the equations below.


$$\mathrm{Fat}\;\mathrm{digestibility}\;of\;\mathrm{diet}\;(\%)=\;[\frac{\mathrm{Fat}\;\mathrm{consumed}-\mathrm{Fat}\;\mathrm{excreted}}{\mathrm{Fat}\;\mathrm{consumed}}]x\;100$$

Where fat consumed (g) = diet intake (g) × fat in diet (%); fat excreted (g) = excreta output (g) × fat in excreta (%). The fat digestibility values for test diets were then calculated by difference using the equation:


Fat digestibility of test diet (%)=Fat digestibility of ground corn reference diet - [(apparent fat digestibility of ground corn reference diet-apparent fat digestibility of test diet mixture with corn)proportion of test diet fat substituted into the test diet mixture with corn]

### Nitrogen-corrected true metabolizable energy (TME_n_) calculations

The calculation of TME_n_ was performed according to Parsons et al. (1982). Correction for endogenous energy excretion was determined using data collected from numerous fasted birds over several years. The TME_n_ values were calculated using the following equation:


TMEn of diet (kcalg)= [GE consumed-(GE excreted by fed birds + 8.22 x nitrogen retained by fed birds)+(GE excreted by fasted birds+8.22 x nitrogen retained by fasted birds)feed intake (g)]

Where GE consumed (kcal) = diet intake (g) × GE of diet (kcal/g); GE excreted by fed or fasted birds (kcal) = excreta output (g) × GE of excreta (kcal/g); 8.22 = GE (kcal) of uric acid per g of nitrogen (Hill and Anderson, 1958); nitrogen retained by fed or fasted birds (g) = diet intake (g) × diet nitrogen (%) – excreta output (g) × excreta nitrogen (%). The TME_n_ values were then calculated by difference, as shown below.


$${\mathrm{TME}}_{n}(\frac{\mathrm{kcal}}{g})=\;{\mathrm{TME}}_{n}\;of\;\mathrm{ground}\;\mathrm{corn}\;\mathrm{reference}\;\mathrm{diet}\;-\;[\frac{{\mathrm{TME}}_{n}\;of\;\mathrm{ground}\;\mathrm{corn}\;\mathrm{reference}\;\mathrm{diet}\;{-\;\mathrm{TME}}_{n}\;of\;\mathrm{test}\;\mathrm{diet}}{\mathrm{proportion}\;of\;\mathrm{test}\;\mathrm{diet}\;\mathrm{substituted}\;\mathrm{into}\;\mathrm{the}\;\mathrm{corn}\;\mathrm{reference}\;\mathrm{diet}}]\;$$

The AA digestibility data derived from the current study were used to estimate the digestible indispensable AA concentrations of the diets tested on a weight basis (g digestible AA/100 g diet). The AA digestibility data and TME_n_ data derived from the current study were used to estimate the digestible indispensable AA concentrations of the diets tested on a caloric basis (g digestible AA/1,000 kcal ME).

### Statistical analyses

Data were analyzed using the Mixed Models procedure of SAS (version 9.4, SAS Institute, Inc., Cary, NC), with the experimental unit being individual roosters. The fixed effect of treatment was tested, and rooster was considered a random effect. Data were tested for normality using the UNIVARIATE procedure of SAS. Differences between treatments were determined using a Fisher-protected least significant difference with a Tukey adjustment to control for experiment-wise error. A probability of *P *< 0.05 was accepted as statistically significant, and *P *< 0.10 was considered a trend. Reported pooled standard errors of the mean were determined according to the Mixed Models procedure of SAS.

## Results

Although all diets were processed from the same raw ingredient batch, there were minor differences in the analyzed nutrient composition (Table 2). Protein ranged from 47.54% to 51.81%, fat ranged from 34.35% to 36.22%, fiber ranged from 5.60% to 10.62%, and ash ranged from 7.21% to 7.91%. Dietary indispensable and dispensable AA concentrations of the diets and AAFCO (2024) minimum dietary indispensable AA concentration recommendations for adult dogs at maintenance are presented in Table 3. All 6 diets had dietary AA concentrations well above those recommended by AAFCO, both on a weight (% AA, DM basis) and caloric (g/1,000 kcal ME) basis. The reactive lysine: total lysine ratios were 0.95, 0.95, 0.93, 0.95, 0.96, 0.96, for the RT, SV, ST, HPP, FD, and RAW diets, respectively (Table 2).

**Table 2.** Analyzed chemical analysis of dog diets tested

Item	RT	SV	ST	HPP	FD	RAW
**Dry matter, %**	30.47	31.33	31.08	32.00	96.66	29.69
	------ DM basis ------					
**Protein, %**	48.18	48.78	47.54	49.67	49.11	51.81
**Acid-hydrolyzed fat, %**	34.35	34.49	35.57	36.22	35.72	34.61
**Total dietary fiber, %**	10.62	5.60	6.57	8.35	7.83	8.06
** Insoluble dietary fiber, %**	8.38	4.20	5.38	5.82	6.69	5.82
** Soluble dietary fiber, %**	2.24	1.40	1.19	2.53	1.14	2.24
**Ash, %**	7.91	7.31	7.82	7.21	7.86	7.69
**Nitrogen-free extract^1^, %**	−1.06	3.82	2.50	−1.45	−0.52	−2.17
**Gross energy, kcal/kg**	6.41	6.39	6.38	6.33	6.32	6.35
**Metabolizable energy^2^, kcal/g**	5.02	5.21	5.20	5.25	5.18	5.19
**Metabolizable energy^3^, kcal/g**	4.61	4.77	4.77	4.82	4.76	4.76
**Reactive lysine: total lysine ratio**	0.95	0.95	0.93	0.95	0.96	0.96

1 Nitrogen-free extract, % = 100 – (acid-hydrolyzed fat {%} + crude protein {%} + ash {%} + TDF {%}).

2 Metabolizable energy estimated using Atwater factors (4 kcal/g for protein and nitrogen-free extract; 9 kcal/g for fat); negative values were changed to 0 before calculation.

3 Metabolizable energy estimated using modified Atwater factors (3.5 kcal/g for protein and nitrogen-free extract; 8.5 kcal/g for fat); negative values were changed to 0 before calculation.

**Table 3.** Indispensable and dispensable amino acid (AA) concentrations of dog foods tested

	% dry matter							g/1,000 kcal^2^						
Item	AAFCO^1^	RT	SV	ST	HPP	FD	RAW	AAFCO	RT	SV	ST	HPP	FD	RAW
**Indispensable AA**														
**Arginine**	0.51	3.35	3.40	3.34	3.31	3.33	3.41	1.28	7.27	7.13	7.00	6.87	7.00	7.16
**Histidine**	0.19	1.45	1.48	1.45	1.41	1.41	1.49	0.48	3.15	3.10	3.04	2.93	2.96	3.13
**Isoleucine**	0.38	2.45	2.50	2.50	2.44	2.38	2.50	0.95	5.31	5.24	5.24	5.06	5.00	5.25
**Leucine**	0.68	4.07	4.14	4.13	4.03	3.98	4.17	1.70	8.83	8.68	8.66	8.36	8.36	8.76
**Lysine**	0.63	4.19	4.10	4.20	4.11	4.03	4.33	1.58	9.09	8.60	8.81	8.53	8.47	9.10
**Methionine**	0.33	1.29	1.34	1.34	1.30	1.28	1.33	0.83	2.80	2.81	2.81	2.70	2.69	2.79
** Methionine + cysteine**	0.65	1.91	2.00	1.99	1.95	1.93	2.01	1.63	4.14	4.19	4.17	4.05	4.05	4.22
**Phenylalanine**	0.45	2.36	2.36	2.40	2.31	2.28	2.43	1.13	5.12	4.95	5.03	4.79	4.79	5.11
** Phenylalanine + tyrosine**	0.74	4.31	4.38	4.34	4.22	4.26	4.50	1.85	9.35	9.18	9.10	8.76	8.95	9.45
**Threonine**	0.48	2.24	2.29	2.28	2.24	2.20	2.30	1.20	4.86	4.80	4.78	4.65	4.62	4.83
**Tryptophan**	0.16	0.50	0.51	0.49	0.45	0.48	0.45	0.40	1.08	1.07	1.03	0.93	1.01	0.95
**Valine**	0.49	2.81	2.80	2.81	2.77	2.75	2.85	1.23	6.10	5.87	5.89	5.75	5.78	5.99
**Dispensable AA**														
**Alanine**	-	3.10	3.13	3.06	3.04	3.05	3.13	-	6.72	6.56	6.42	6.31	6.41	6.58
** Aspartic acid**	-	4.61	4.69	4.66	4.59	4.55	4.69	-	10.00	9.83	9.77	9.52	9.56	9.85
**Cysteine**	-	0.62	0.66	0.65	0.65	0.65	0.68	-	1.34	1.38	1.36	1.35	1.37	1.43
** Glutamic acid**	-	6.86	7.03	6.88	6.85	6.88	6.97	-	14.88	14.74	14.42	14.21	14.45	14.64
**Glycine**	-	3.11	3.07	2.95	3.01	3.06	3.07	-	6.75	6.44	6.18	6.24	6.43	6.45
**Proline**	-	2.46	2.43	2.42	2.42	2.47	2.46	-	5.34	5.09	5.07	5.02	5.19	5.17
**Serine**	-	1.90	2.00	1.94	1.96	1.94	1.98	-	4.12	4.19	4.07	4.07	4.08	4.16
**Tyrosine**	-	1.95	2.02	1.94	1.91	1.98	2.07	-	4.23	4.23	4.07	3.96	4.16	4.35
**Taurine**	-	0.30	0.29	0.27	0.26	0.28	0.32	-	-	-	-	-	-	-

1 Association of American Feed Control Officials (AAFCO, 2024) for adult dogs at maintenance.

2 Calculated with modified Atwater values.

On a numerical basis, the RAW diet had the highest and the RT diet had the lowest AA digestibility (Table 4). However, AA digestibility was very high for all diets, with all indispensable AA having coefficients >90%. The only exception was the histidine digestibility (87.79%) for the HPP diet. The standardized histidine digestibility was greater (*P *< 0.05) for the SV (95.12%) and ST (95.53%) diets than all other diets (87.79–90.80%). The standardized aspartic acid digestibility was greater (*P *< 0.05) for the ST (95.00%), HPP (93.96%), FD (95.00%), and RAW (95.71%) diets than the RT diet (89.18%), with SV being intermediate (92.88%). Valine, methionine, leucine, isoleucine, and phenylalanine digestibility tended to be higher (*P *< 0.10) for the RAW diet than for the RT diet. Additionally, isoleucine digestibility tended to be greater (*P *< 0.10) for the ST diet than the RT diet. The TME_*n*_ values of the SV (5.26 kcal/g DM) and ST (5.26 kcal/g DM) diets were greater (*P *< 0.05) than that of the RT (4.81 kcal/g DM), HPP (4.81 kcal/g DM), and FD (4.80 kcal/g DM) diets with the RAW diet being intermediate (5.07 kcal/g DM). TME_*n*_ did not correlate with AA digestibility data and is not entirely explained by numerical differences in fat digestibility because the RAW diet had the second highest numerical fat digestibility, but the SV diet had a higher numerical TME_*n*_ than the RAW diet.

**Table 4.** Amino acid (AA) and fat digestibility and nitrogen-corrected true metabolizable energy (TME_n_) of dog diets using cecectomized and conventional rooster assays

Item	RT	SV	ST	HPP	FD	RAW	SEM	*P*-value
**Indispensable AA, %**								
Arginine	95.14	96.05	97.17	96.53	97.44	97.70	0.920	0.3856
Histidine	90.80^b^	95.12^a^	95.53^a^	87.79^b^	90.58^b^	90.07^b^	0.891	<0.0001
Isoleucine	92.40	95.33	96.54	95.06	95.97	96.61	1.050	0.0866
Leucine	92.77	95.62	96.73	95.74	96.66	97.36	1.114	0.0944
Lysine	92.50	94.34	96.21	94.75	95.42	95.48	0.972	0.1596
Methionine	93.86	95.69	96.76	96.04	96.69	97.07	0.768	0.0730
Phenylalanine	91.53	94.37	95.56	94.62	95.26	96.23	1.120	0.0933
Threonine	91.30	94.42	95.96	94.07	95.38	95.74	1.452	0.2551
Tryptophan	97.21	97.14	97.64	98.88	97.70	99.10	0.940	0.5576
Valine	90.31	93.13	94.75	94.11	95.37	96.01	1.307	0.0633
**Dispensable AA, %**								
Alanine	92.49	94.26	95.76	94.18	96.18	94.18	1.129	0.1701
Aspartic acid	89.18^b^	92.88^ab^	95.00^a^	93.96^a^	95.00^a^	95.71^a^	1.063	0.0027
Cysteine	77.94	86.94	88.57	82.36	86.14	89.17	3.489	0.2242
Glutamic acid	92.35	94.61	95.68	94.41	95.60	96.04	1.016	0.1580
Proline	94.46	95.75	97.16	91.68	93.89	94.08	1.487	0.2122
Serine	88.33	93.40	94.38	92.50	93.71	94.67	1.835	0.1928
Tyrosine	89.73	92.60	93.59	92.22	93.59	94.44	1.227	0.1478
Fat digestibility, %	87.89	90.54	92.28	87.96	85.69	92.22	2.051	0.1740
TME_n_ ^1^, kcal/g	4.81^b^	5.26^a^	5.26^a^	4.81^b^	4.80^b^	5.07^ab^	0.111	0.0074
TME_n_/GE^1^, %	75.10^b^	82.39^a^	82.54^a^	76.06^b^	75.95^b^	79.82^ab^	1.737	0.0114

1 Significant differences among treatments, but not after multiple comparisons. Means lacking a common superscript differ without Tukey adjustment (*P *< 0.05).

ab Within a row, means lacking a common superscript differ (*P *< 0.05).

Using the AA digestibility data and the TME_*n*_ data from the present study, numerical estimates of digestible AA on a weight (g AA/100g DM diet) and caloric (g AA/1,000 kcal ME) basis were calculated (Table 5). On a weight basis, the RAW diet had the highest digestible AA concentrations for all indispensable AA, except for histidine (SV), methionine (ST), and tryptophan (SV). Additionally, the RT diet had the lowest digestible AA concentrations for all indispensable AA, except for histidine (HPP), lysine (FD), and tryptophan (HPP). On a caloric basis, the RT diet had the highest digestible histidine and tryptophan concentrations and the lowest digestible methionine + cysteine concentrations. The HPP diet had the highest digestible isoleucine, leucine, methionine, phenylalanine, and threonine concentrations and the lowest digestible histidine concentrations. The FD diet had the highest digestible arginine, leucine, methionine + cysteine, and valine concentrations. The RAW diet had the highest digestible lysine and phenylalanine + tyrosine concentrations and the lowest digestible tryptophan concentrations. The SV diet had the lowest digestible isoleucine, leucine, lysine, methionine, phenylalanine, phenylalanine + tyrosine, threonine, and valine concentrations. The ST diet had the lowest digestible arginine concentrations.

**Table 5.** Digestible indispensable amino acid concentrations of dog foods tested^1^

	g/100 g DM of diet						g/1,000 kcal of diet					
Item	RT	SV	ST	HPP	FD	RAW	RT	SV	ST	HPP	FD	RAW
Arginine	3.19	3.27	3.25	3.20	3.24	3.33	6.62	6.21	6.16	6.64	6.76	6.57
Histidine	1.32	1.41	1.39	1.24	1.28	1.34	2.73	2.68	2.63	2.57	2.66	2.65
Isoleucine	2.26	2.38	2.41	2.32	2.28	2.42	4.70	4.53	4.58	4.82	4.76	4.76
Leucine	3.78	3.96	3.99	3.86	3.85	4.06	7.84	7.52	7.59	8.02	8.02	8.01
Lysine	3.88	3.87	4.04	3.89	3.85	4.13	8.05	7.35	7.68	8.09	8.02	8.15
Methionine	1.21	1.28	1.30	1.25	1.24	1.29	2.52	2.44	2.46	2.59	2.58	2.55
Methionine + cysteine	1.69	1.86	1.87	1.78	1.80	1.90	3.52	3.53	3.56	3.71	3.75	3.74
Phenylalanine	2.16	2.23	2.29	2.19	2.17	2.34	4.49	4.23	4.36	4.74	4.53	4.61
Phenylalanine + tyrosine	3.91	4.10	4.11	3.95	4.03	4.29	8.12	7.79	7.81	8.20	8.39	8.47
Threonine	2.05	2.16	2.19	2.11	2.10	2.20	4.25	4.11	4.16	4.38	4.37	4.34
Tryptophan	0.49	0.50	0.48	0.44	0.47	0.45	1.01	0.94	0.91	0.92	0.98	0.88
Valine	2.54	2.61	2.66	2.61	2.62	2.74	5.27	4.96	5.06	5.42	5.47	5.40

1 Using the AA digestibility and TME_n_ data from the current study.

## Discussion

It is thought that raw or mildly cooked pet foods have greater AA digestibility than conventional (extrusion or retort) formats (Geary et al., 2023) because of the less intensive processing applied, particularly regarding heat, which can decrease AA digestibility (Hurrell and Finot, 1985; Hendriks et al., 1999; Williams et al., 2006; Tran et al., 2008). However, these alternative types of pet food are often formulated with different ingredient profiles and macronutrient targets, making it challenging to determine what drives digestibility changes. To isolate the impacts of various common commercial dog food processing procedures on AA digestibility and the caloric content of pet foods, the current study tested 6 diets made from the same raw batch but processed in different ways, including the raw batch, which had no pre-processing applied.

Although there were minimal significant statistical differences in AA digestibility, there were 5 trends of the RAW diet having greater indispensable AA digestibility than the RT diet. These findings are similar to a study conducted by Oba et al. (2019), in which AA digestibilities were determined for chicken meal, retorted chicken, steamed chicken, and raw chicken using the cecectomized rooster model (with substrates similarly freeze-dried before crop intubation). There were no statistical differences, but the raw chicken had numerically higher indispensable AA digestibility than the retorted chicken (Oba et al., 2019). However, Oba et al. (2023) showed no AA digestibility differences between FD or frozen raw diets, similar to the results of the present study. Agreeing with the results of the current study, in Oba et al. (2019) steamed chicken had significantly higher histidine digestibility than the raw chicken. However, the steamed chicken had numerically higher AA digestibility for all indispensable AA than the raw chicken in Oba et al. (2019), which was not consistently observed in the present study. The few statistical differences suggest these processing methods may not have large impacts on AA digestibility. It is unclear why the histidine digestibility increased with mild cooking, but no other AA had these same trends. In Geary et al. (2023), commercial pet foods of various diet formats were analyzed for AA digestibility, also using the cecectomized rooster model. The mildly cooked diet did not have higher histidine digestibility than the other diet formats (Geary et al., 2023). The reactive lysine: total lysine ratios, an indicator of heat damage, did not correlate with the digestibility data (were consistent for all diets) even though the decrease in digestibility is likely due to heat damage. However, measuring advanced glycation end-products may be more informative of heat damage in the diets than the reactive lysine: total lysine ratios (van Rooijen et al., 2014).

The TME_n_/GE of the RT diet (75.10%) was much lower than retorted chicken (82.68%) measured in a prior study (Oba et al., 2019). However, the previous study only analyzed chicken, while the present study analyzed complete dog diets, making comparisons difficult. However, in Oba et al. (2019), the retorted chicken TME_n_ was not different from that of the steamed (5.34 kcal/g) and raw (5.92 kcal/g) chicken, whereas, in the present study, the ST diet (5.26 kcal/g) had a higher TME_n_ than the RT diet. However, the RAW diet (79.82%) was lower than the raw chicken evaluated (84.83%) (Oba et al., 2019). Steamed chicken (81.05%; Oba et al., 2019) had a comparable TME_n_/GE to the mildly cooked diets measured in the present study (SV: 82.39%; ST: 82.54%).

In a study that calculated ME by both calculated and measured (via bomb calorimetry) food and fecal energy content, the mildly cooked chicken diet also had a comparable ME/GE (86.4%) to the present study (Tanprasertsuk et al., 2021). The TME_n_/GE in the present study, however, was lower than the mildly cooked chicken-based diets in Geary et al. (2023) (92.22%) and in Oba et al. (2020) (91.50%). The TME_n_/GE of the RAW diet (79.82%) in the present study was much lower than a raw chicken-based diet (91.56%) analyzed in Geary et al. (2023) but comparable to a raw chicken-based diet (82.07%) analyzed in Oba et al. (2023). The FD diet evaluated in the present study had a slightly lower TME_n_/GE ratio (75.95%) than the FD chicken-based diets analyzed in Geary et al. (2023) (82.70%) and Oba et al. (2023) (82.07%). As processing type does affect TME_n_, as evidenced by the current study, differences across studies might be due to the specific processing parameters or ingredient inclusions. The estimated metabolizable energy using Atwater factors predicted the true metabolizable energy of the mildly cooked diets well; although, the TME of the other diet formats fell between the estimates using the modified Atwater and Atwater factors. These results demonstrate the inaccuracy of using modified Atwater factors for mildly cooked and raw foods, with even high-quality conventionally manufactured diets being poorly estimated using those factors.

Unexpectedly, the TME_n_ did not correlate well with the AA digestibilities. Determining fat digestibility with the conventional excreta provided insight as to why the ST and RAW diets had higher TME_n_, but it is not as clear for the SV diet because while it was numerically higher than the RT, HPP, and FD diets, it was not as numerically high as ST and RAW. As the diet contained approximately 50% protein and 35% fat, these 2 macronutrients are the most significant drivers of ME. Considering the total digestible AA, RAW had the highest, followed by ST, SV, FD, HPP, and RT. Total digestible fat followed a slightly different pattern, with ST being highest and followed by RAW, HPP, SV, FD, and RT. Combining these 2 explained why the ST diet had a higher TME_n_ but not the SV diet. As calculated, the mildly cooked diets are the only diets with a non-negative nitrogen-free extract value. Even if these negative values are assumed to be zero, the additional ME from the carbohydrates in the mildly cooked diets completes the explanation for why these diets have a higher TME_n_. However, all diets were formulated with the same level of carbohydrates, so the carbohydrates might be more digestible in the mildly cooked diets. Furthermore, carbohydrate digestibility should be high for all the cooked diets. Alternatively, another component, such as protein, fat, or ash, could be double-analyzing as that macronutrient and fiber, falsely elevating the TDF for all diets, but most especially the raw and RT diets. For example, collagen can undergo hydrolysis of its hydrogen bonds when heated at a low temperature, but can gelatinize at higher temperatures (Tornberg, 2005; Dominguez-Hernandez et al., 2018). Therefore, the mildly cooked diets (SV and ST) may have had more digestible collagen, leading to an increased ME value. Additionally, the cartilage in the chicken neck may also have been analyzed as fiber, as chondroitin sulfate and hyaluronic acid have fiber-like structures. Although each diet should have the same levels of cartilage, the amount being able to be digested by both the roosters and the TDF enzymes may have been influenced by processing conditions.

There were a few limitations to this study, including the fact that this study aimed to delineate differences in AA digestibility and TME_n_ due to diet processing; however, all wet diets (RT, SV, ST, HPP, RAW) had to be freeze-dried in order to be successfully crop intubated into the roosters. Therefore, instead of comparing retorted, sous vide cooked, steamed, high-pressure processed, freeze-dried, and raw dog foods as-is, this experiment tested freeze-dried retorted, freeze-dried sous vide cooked, freeze-dried steamed, 2 freeze-dried diets with high-pressure processing (although with different temperature and time parameters for freeze-drying), and one freeze-dried diet without high-pressure processing. However, because the same freeze-drying procedure was applied to all of the wet diets, changes between these diets may be attributable to the processing pre-freeze-drying. Though, due to their differences in prior processing, there could be variances in how the secondary freeze-drying affected the diets. Additionally, during initial processing, the SV, ST, and HPP diets were all processed in the same packaging size (4.55 kg chubs); however, due to size restrictions for the RT equipment and efficiency in achieving low water activity, the RT diet was processed in 200 g pouches, and the FD diet was processed in 1.95 cm^3^ cubes, respectively. The SV and ST diets would have needed less processing time if processed in smaller amounts, which might have influenced AA digestibility. Also, we noticed that there was much higher variability observed in roosters when fed these diet formats compared with previous studies testing extruded diets or single ingredients. Using only 5 roosters per diet might have reduced our power to detect many differences. With high variability, a relatively low effect size, and a small sample size, the ability to detect differences was diminished. Because a Tukey adjustment was used to reduce the risk of Type I error, power was further reduced and with the small sample size, the risk of a Type II error was increased.

In conclusion, all diet formats had indispensable AA concentrations well above the AAFCO guidelines. Additionally, all diet formats had high AA digestibility, with all indispensable AA above 90%, with the exception of HPP for histidine (87.79%). There were minimal differences in AA digestibility among diets. These results suggest processing might not have a large effect on amino acid digestibility. The TME_n_ was higher in the mildly cooked diets (SV and ST) compared with other diet formats but not different from that of the RAW mix. On a weight basis, the RAW mix had the greatest quantity (7/10) of the highest digestible indispensable AA concentrations while RT had the highest number (7/10) of the lowest concentrations. On a caloric basis, the HPP diet had greatest quantity (5/10) of the highest digestible indispensable AA concentrations, while the SV diet had the highest number (7/10) of the lowest concentrations. Although processing did influence AA digestibility in this study, the differences among these diet formats and nutrient concentrations were minor and may not be physiologically relevant. Diets containing other ingredient profiles and nutrient concentrations should also be tested to determine whether these factors lead to greater differences.

## Abbreviations

AA: amino acid
AAFCO: Association of American Feed Control Officials
AOAC: Association of Official Analytical Chemists
DM: dry matter
FD: freeze-dried raw diet
HPP: high pressure processed raw diet
ME: metabolizable energy
RT: retorted diet
ST: steamed diet
SV: sous vide diet
TME_n_: nitrogen-corrected true metabolizable energy
